# Operations for Empyema, Particularly Their After-Treatment

**Published:** 1889-04

**Authors:** Hugo Holsti, Herman Mynter

**Affiliations:** Helsingfors; Buffalo, N. Y., Professor of Surgery, Medical Department, Niagara University


### Translations.

"OPERATIONS FOR EMPYEMA, PARTICULARLY THEIR AFTER-TREATMENT. BY PROF. HUGO HOLSTI, OF HELSINGFORS (ARCHIV. FUER KLINISCHE MEDICIN, NO. 42, 1886)—" SHOULD THE PLEURAL CAVITY BE WASHED OUT." BY M. M. BASIL (MED. CHRONICLE, AUGUST, 1887) — "WHEN IS INCISION TO BE AVOIDED IN PURULENT PLEURITIC EXUDATIONS"? BY PROF. O. FRAENTZEL (CHARITE ANNALS, 1888). TRANSLATED FROM THE *Norwegian Medicinsk Revue*.

BY HERMAN MYNTER, M. D., Buffalo, N. Y.

Professor of Surgery, Medical Department, Niagara University.

Most authors seem at present to agree that an empyema may only exceptionally be absorbed by Nature's own help, that no medical treatment has any effect, and that we do not definitely cure a patient by puncture, with or without aspiration, at least not in adults, but that the radical treatment, a thoracic fistula, becomes necessary.

About the operation itself there is still some disagreement, but everything seems to indicate that resection of a costa, with subsequent opening of the pleural cavity in the lateral region, will be considered the most proper operation.

There are still different opinions about the after-treatment. Bartels and Kussmaul use daily irrigation, which was used in Helsingfors till 1883. The results were not satisfactory, particularly with regard to the permanent fistula, and Professor Runeberg commenced, therefore, after 1883, to use the method recommended by König, Wagner and others, to irrigate the cavity once immediately after the operation. This method was given up from the Fall of 1885, and in seventeen cases since treated no irrigation has been used. The occasion for the change was a case in which a communication with the bronchi was evidently present. The irrigation was omitted in order not to expose the bronchi to the irritating effect of antiseptic fluids. The case terminated favorably, and the stinking pus lost, in a few days, its fetid character.

The author has treated twenty-seven cases, of which ten were irrigated once, immediately after the operation. Of these, one died, a man sixty-three years of age, who suffered from valvular disease of the aorta, and died of pneumonia; two left with a permanent fistula, and seven recovered. Of the seventeen treated without irrigation, one left in five weeks (on his own demand) with a fistula not perfectly healed; all the others recovered. Recovery occurred in an average of seventy-one days after the operation; the shortest time was five and one-half weeks; the longest, five and one-half months.

Most authors are inclined to believe that the longer the empyema has lasted before the operation, the less prospect is there of an early recovery. The author does not agree with this statement. Of the twenty-three perfectly cured patients, nine were operated prior to one month's duration of the disease. Of these, only three were cured in the course of two months; with the others it took longer time—in two cases respectively, 157 and 166 days. If the disease had existed two to three, or even five to six months, the recovery took place earlier. With a duration of from nine months to several years, the recovery took longer time.

The patient's age may have some influence on the duration of the convalescence, which probably is shortest in childhood. The average duration in nine patients operated in 1884 and 1885, was thirty-eight days; in fourteen patients in 1886, sixty days, and in the last seven, forty-five days. It looks, therefore, as if the duration had gradually been diminished, and as if the omission of irrigation, to say the least, had had no unfavorable influence on the recovery. Complications, such as communications with the bronchi and fistulas, have had no evident influence on the duration. When tuberculosis was present, no operation, save a simple puncture, has been performed.

The author does not consider it improbable that repeated irrigations may occasion a permanent fistula. Even if done carefully, it cannot be avoided, that thin, commencing adhesions are torn over, by which the growing together of the opposite layers of the pleura is made more difficult. Carbolic acid, besides, has a bad influence on the process of granulation. In most cases of empyema, it is difficult to show the cause; once in a while it may be a trauma, but it is, probably, oftenest the result of a pneumonia. Both are found most frequently on the right side. The operation has generally been made in the lateral region, between the axillary and mammilary line, where there is less of muscular tissue, and where the operation may be done without placing the patient on the healthy side, which often may occasion a dangerous attack of dyspnea. It has been argued that retention of pus occurs easier with the fistula here than on the back.

This does not agree with the author's experience. He recommends, to be sure, that the patient lie on the operated side as much as possible, which, as a rule, is done without difficulty.

If the collection of pus is circumscribed, the fistula, of course, must be made where it is necessary. The operation is always combined with the resection of the sixth costa, sometimes, besides, of the fifth and seventh. We gain by that a better drainage without prolonging the operation or making it much more difficult. The fear of deformity of the thorax, as a result of the operation, is only of a theoretical nature. The operation is easy to make. Sometimes, in going through the pleura, some bleeding may occur. It may be parenchymatous, from the newly-formed vessels in the pleura, and need not indicate that the intercostal arteries were injured. If this should happen, it may become necessary to plug the opening with sublimate-gauze for a day. After the pleural cavity has been opened, it ought to be evacuated slowly, to avoid too quick changes in the intra-thoracic pressure.

The most important thing in the after-treatment is to make provision for perfect evacuation of pus. This may be difficult if the fistula becomes so narrow that no drainage-tube can be introduced, while there still is found a pus-secreting cavity behind. It is therefore necessary, if fever should occur, to examine the condition of the fistula and, possibly, make a counter-opening. A question of importance in regard to the after-treatment is, when the drainage-tube ought to be removed. It ought not, as a general rule, to be removed too early. We must convince ourselves, as near as possible, that no cavity exists behind the fistula, which is best done with a long, flexible, well-disinfected probe; for instance, a uterine probe. Being in this way convinced that no cavity longer exists, the drainage-tube may be removed.

It happens quite frequently that the cavity ceases to decrease in size. This may be found out by injecting a weak solution of corrosive sublimate. The amount of fluid used indicates the size of the cavity. If the cavity ceases to decrease, injection of tincture of iodine may stimulate the process of granulation. If we do not succeed by this method, we may have to resort to extensive resections of costæ. M. Basil has mostly the same opinion as Professor Holsti, with the difference that he, as a rule, irrigates the cavity once, immediately after the operation, particularly if fibrinous pieces and coagula be present, also in hopeless cases, to prevent decomposition of pus. But he considers irrigation of the pleural cavity in adults a dangerous proceeding, which ought to be performed in each case only on special indications.

Reporting some cases of suddenly occurring death during irrigation, he calls attention to the fact that this has never happened by a first irrigation, but only by oft-repeated ones. It has made no difference what fluid was used, or which side was affected.

In studying the cause of death, and the dangerous symptoms, the author first treats of those cases of death which occur by pleuritic exudations, taken as a whole. The causes, according to him, are: *First*, edema in the other lung; *second*, paralysis of the center of respiration in the medulla oblongata (sudden change of position and turning around of the patient on the healthy side may act predisposing); *third*, partial or total closure of vena cava inferior; *fourth*, changes in the muscles of the heart, and in the valves; *fifth*, thrombosis or emboli in the arteries of lung, brain, or heart, or sudden anemia of the brain; *sixth*, too great dilatation of the ventricle, occasioned by sudden evacuation of the pleural cavity, or pressure on, respectively displacement of, the heart by sudden exudation into the pleural cavity; *seventh*, paralysis of the heart, either primarily or from reflex causes; nerve-branches are found in the pleural cavity, intimately connected with the vagus; besides, we are very near the ganglia of the heart and the sympathetic nerve, both in the thoracic and abdominal cavity, also closely connected with the ganglia of the heart; *eighth*, the function of the heart may be disturbed by the physiological action of chlorine-water, sulphuric acid, opium, acetic acid, etc., when applied on the heart itself or in its neighborhood; *ninth*, some inexplainable cases of sudden death by exudations into the pleural cavity are always left; they are analogous to those occurring in ascites, ovarian tumors, opening of large abscesses, etc.

As irrigating fluids, he recommends weak solutions of boracic acid, creosote, corrosive sublimate (1 to 8,000, or 1 to 6,000, if the condition of the patient be such that no danger would occur from a possible diarrhea). Chlorine-salts and acetic acid must not be used, as they act depressingly on the heart, nor strongly-stimulating fluids, which irritate the nerves; even tincture of iodine in warm water (1 to 30) ought to be avoided. The irrigation must be done by the physician himself with low pressure, and must not be too voluminous. The direction of the stream, and the temperature of the fluid must be under control. As a prophylactic against reflex paralysis of the heart, the author recommends, from theoretical reasons, atropine subcutaneously.

In regard to the indications for the operation, the author gives the rule: to resect and drain in cases of old empyema; and to make the resections oftener and in the greatest possible extension. The dangers are not great. Failure occurs when the resections have not been extensive enough. Adhesions, membranes, etc., which prevent the dilatation of the lung, must be torn over or scraped away. Basil does not mention any contra-indications for operation, but Holsti considers progressive tuberculosis a contra-indication, and limits himself in such cases to puncture. This agrees with the opinion of Professor Leyden and Professor Fräntzel. Even if we do not find tuberculous bacilli in the sputum or exudation, and the lungs do not show physical signs of phthisis, we are able to judge about the specific character of the exudation, when we in a young fever-free individual meet with a large exudation, which perfectly returns in a few days after aspiration. In such cases, even repeated punctures of a serous exudation is contra-indicated, as the patient loses strength by it.

Professor Fräntzel condemns the operation in empyemas, in which the wall of the thorax is stiff and unyielding, and in which we, *a priori*, may know that the lung on the diseased side will be unable to dilate. In such cases the wound, after even the most extensive resections, will never heal properly.

He advises against operation, too, in purulent exudations, when there is no fever and the patient is old. Not rarely we find in such cases malignant new growths, and the patient lives longer if left alone.

